# Activator Protein-1 (AP-1) Signaling Inhibits the Growth of Ewing Sarcoma Cells in Response to DNA Replication Stress

**DOI:** 10.1158/2767-9764.CRC-23-0268

**Published:** 2023-08-17

**Authors:** Emma E. Croushore, Stacia L. Koppenhafer, Kelli L. Goss, Elizabeth L. Geary, David J. Gordon

**Affiliations:** 1 Department of Pediatrics, Division of Pediatric Hematology/Oncology, University of Iowa, Iowa City, Iowa.

## Abstract

Ribonucleotide reductase (RNR) catalyzes the rate-limiting step in the synthesis of deoxyribonucleosides and is required for DNA replication. Multiple types of cancer, including Ewing sarcoma tumors, are sensitive to RNR inhibitors or a reduction in the levels of either the RRM1 or RRM2 subunits of RNR. However, the polypharmacology and off-target effects of RNR inhibitors have complicated the identification of the mechanisms that regulate sensitivity and resistance to this class of drugs. Consequently, we used a conditional knockout (CRISPR/Cas9) and rescue approach to target RRM1 in Ewing sarcoma cells and identified that loss of the RRM1 protein results in the upregulation of the expression of multiple members of the activator protein-1 (AP-1) transcription factor complex, including c-Jun and c-Fos, and downregulation of c-Myc. Notably, overexpression of c-Jun and c-Fos in Ewing sarcoma cells is sufficient to inhibit cell growth and downregulate the expression of the c-Myc oncogene. We also identified that the upregulation of AP-1 is mediated, in part, by SLFN11, which is a replication stress response protein that is expressed at high levels in Ewing sarcoma. In addition, small-molecule inhibitors of RNR, including gemcitabine, and histone deacetylase inhibitors, which reduce the level of the RRM1 protein, also activate AP-1 signaling and downregulate the level of c-Myc in Ewing sarcoma. Overall, these results provide novel insight into the critical pathways activated by loss of RNR activity and the mechanisms of action of inhibitors of RNR.

**Significance:**

RNR is the rate-limiting enzyme in the synthesis of deoxyribonucleotides. Although RNR is the target of multiple chemotherapy drugs, polypharmacology and off-target effects have complicated the identification of the precise mechanism of action of these drugs. In this work, using a knockout-rescue approach, we identified that inhibition of RNR upregulates AP-1 signaling and downregulates the level of c-Myc in Ewing sarcoma tumors.

## Introduction

Ribonucleotide reductase (RNR), which is composed of RRM1 and RRM2 subunits, catalyzes the rate-limiting step in the synthesis of deoxyribonucleotides and is required for DNA replication and DNA damage repair (1). Inhibition of RNR causes DNA replication stress and both subunits of this enzyme complex are the targets of multiple chemotherapy drugs (1–4). Inhibitors of RNR, which include gemcitabine, clofarabine, triapine, and hydroxyurea, are used in clinical oncology to treat a variety of different types of cancer (4, 5). For example, clofarabine and hydroxyurea are used to treat leukemia and gemcitabine is used for the treatment of pancreatic, breast, ovarian, and lung cancers. Gemcitabine is also used in combination with taxane drugs for the treatment of relapsed sarcomas (6).

However, despite the active clinical use of RNR inhibitors, polypharmacology and off-target effects have complicated the identification of the precise mechanism of action of these drugs (1, 5, 7, 8). For example, gemcitabine is an irreversible inhibitor of the RRM1 subunit of RNR, but this pyrimidine nucleoside analog is also incorporated into DNA where it inhibits DNA synthesis and causes masked chain termination and DNA damage (9–11). Similarly, iron chelators, such as triapine, inhibit the function of the RRM2 subunit of RNR, but also target multiple iron-dependent enzymes involved in DNA synthesis and DNA repair (12). Consequently, despite the active clinical use of RNR inhibitors for several decades, the mechanism of how this class of drugs, as both single agents and in combination therapies, induces toxicity in cancer cells is poorly understood (1, 4, 5, 9). This incomplete understanding of the critical mechanism(s) of action of RNR inhibitors, similar to other drugs, has restricted efforts to maximize drug efficacy, identify synergistic drug combinations, and minimize side effects (13–17).

We and others have shown that Ewing sarcoma tumors are sensitive to small-molecule inhibitors of RNR, as well as knockdown of either the RRM1 or RRM2 subunit (18–22). In the current study, we used a conditional knockout (CRISPR/Cas9) and rescue approach to target RRM1 in Ewing sarcoma cells and identify downstream pathways impacted by the loss of RNR activity. Consistent with our previous work and the well-described role of RNR in the biosynthesis of deoxyribonucleotides and its essential role in DNA replication, a reduction in the level of the RRM1 protein caused cell-cycle arrest, DNA damage, and cell death (1). However, transcriptome and gene set enrichment analysis (GSEA) also identified that a decrease in RNR activity in Ewing sarcoma cells results in the upregulation of c-Jun and c-Fos, which are members of the activator protein-1 (AP-1) transcription factor family, and the downregulation of the c-Myc oncogene (23, 24). In addition, we show that the upregulation of AP-1 signaling is mediated in part by SLFN11, a critical regulator of the cellular response to DNA replication stress (25).

Notably, inducible expression of c-Jun and c-Fos in Ewing sarcoma cells inhibits cell growth and downregulates the expression of c-Myc. Similarly, small-molecule inhibitors of RNR, including gemcitabine and hydroxyurea, upregulate expression of c-Jun and c-Fos and downregulate c-Myc in Ewing sarcoma cells. Furthermore, we show that histone deacetylase (HDAC) inhibitors decrease the level of the RRM1 subunit in Ewing sarcoma cells, increase expression of c-Jun, and decrease the expression of c-Myc. Overall, these results provide novel insight into the mechanistic basis of RNR inhibitor toxicity and link increased AP-1 signaling to the regulation of c-Myc levels in Ewing sarcoma tumors.

## Materials and Methods

### Cell Lines and Culture

Cell lines were maintained at 37°C in a 5% CO_2_ atmosphere. The EW8, TC71, TC32, A673, CADO, and SKNEP cell lines were provided by Dr. Kimberly Stegmaier (Dana-Farber Cancer Institute, Boston, MA). The HEK-293T (RRID:CVCL_0045) and U2OS (RRID:CVCL_0042) cell lines were obtained from ATCC. The CHLA-9 and CHLA-10 cell lines were provided by the Childhood Cancer Repository (Children's Oncology Group). The cells were grown in DMEM supplemented with 10% FBS, 100 IU/mL penicillin, and 100 μg/mL streptomycin. Cell lines were used within 8–10 passages after thawing. DNA fingerprinting was used to authenticate cell lines. All cell lines are *Mycoplasma* negative and tested every 6 months for contamination (Universal Mycoplasma Detection Kit, ATCC 30-1012K).

### Chemical Compounds

Chemical compounds were purchased from Thermo Fisher Scientific (puromycin, doxycycline, and geneticin), Sigma (gemcitabine, hydroxyurea, and luciferin), MedChemExpress (fimepinostat, panobinostat, and romidepsin), APExBIO (berzosertib), and Selleckchem (prexasertib).

### Genomics of Drug Sensitivity Data Analysis

Data from the Genomics of Drug Sensitivity in Cancer resource (https://www.cancerrxgene.org/; RRID:SCR_011956) were used to correlate gemcitabine sensitivity data with molecular features associated with drug sensitivity and resistance (26).

### Fluorescent Cell-cycle Reporter

A lentiviral (pLV) plasmid expressing the PIP-FUCCI fluorescent indicator protein construct was obtained from VectorBuilder (27). Lentivirus was prepared as described in previous publications and fluorescent cells were isolated using flow cytometry (Becton Dickinson FACS Aria; ref. 18). Cells were imaged using an EVOS M5000 fluorescent microscope (Thermo Fisher Scientific).

### TO-RRM1-KO Cell Lines

A codon-optimized RRM1 gene construct was obtained as a gene block (IDT) and inserted into the Lenti-X-Tet-One vector (Takara Biology) using NEBuilder HiFi DNA Assembly (NEB). After verification by sequencing, the plasmid was used to make lentivirus, as described in previous publications. CRISPR/Cas9-mediated knockout of RRM1 was then performed using a pLV[CRISPR] plasmid (VectorBuilder) that coexpresses Cas9, a guide RNA (GTGAGTTGTATTCGGGCTAC) targeting *RRM1*, and a hygromycin resistance gene. Lentivirus was prepared as described in previous publications and cells were selected in 400 mg/mL hygromycin starting 48 hours after transduction. The knockout cell lines were then single cell cloned using flow cytometry (Becton Dickinson FACS Aria). Knockout of RRM1 in multiple clones was then validated using immunoblotting.

### Cell Viability Assay

Cell viability was measured using the AlamarBlue (resazurin) fluorescence assay, as described in previous publications (28). Approximately 5,000 cells were plated in each well of a 96-well plate. The next day the cells were treated with a range of drug concentrations for 72 hours. Fluorescence readings were then obtained after adding AlamarBlue (Sigma) using a FLUOstar Omega microplate reader (BMG Labtech). IC_50_ values were calculated using log-transformed and normalized data (GraphPad Prism 9).

### Protein Isolation and Immunoblotting

Whole-cell extracts for immunoblotting were prepared by incubating cells in RIPA buffer (Boston BioProducts) plus protease and phosphatase inhibitors (Halt Protease & Phosphatase Inhibitor Cocktail, Ethylenediaminetetraacetic acid (EDTA)-free; Thermo Fisher Scientific) for 20 minutes. Supernatants were collected following a 15-minute centrifugation at 17,000 r.c.f. at 4°C. Protein loading for the immunoblots was normalized using cell number. SDS-PAGE was used to separate proteins, which were then transferred to polyvinylidene difluoride membranes (Millipore). Nuclear and cytoplasmic fractionation was performed using NE-PER reagents (Thermo Fisher Scientific) according to manufacturer's instructions. Antibodies to the following proteins were used in the immunoblots: c-Jun (60A8, Cell Signaling Technology, #9165, 1:1,000; RRID:AB_2130165), c-Jun (ProteinTech, #10024-2, 1:2,000; RRID:AB_2129714), FLI1 (Santa Cruz Biotechnology #sc-356, 1:1000; RRID:AB_2106116), phospho-Jun (Ser73, ProteinTech #2889-1-AP, 1:1,000; RRID:AB_2881228), c-Fos (Santa Cruz Biotechnology #sc-271243, 1:100; RRID:AB_10610067), phospho-Histone H2A.X (Ser139, Cell Signaling Technology, #9718, 1:1,000; RRID:AB_2118009), PARP (Cell Signaling Technology, #9532, 1:1,000; RRID:AB_659884), RRM1 (Cell Signaling Technology, #8637, 1:1,000; RRID:AB_11217623), RRM2 (Santa Cruz Biotechnology, #398294, 1:500; RRID:AB_2894824), SLFN11 (Santa Cruz Biotechnology, sc-374339; RRID:AB_10989536), Lamin A/C (Developmental Studies Hybridoma Bank, #MANLAC1, 1:100; RRID:AB_2618203), phospho-Chk1 (Ser345, Cell Signaling Technology, #2348, 1:1,000; RRID:AB_331212), Chk1 (Cell Signaling Technology, #2360, 1:1,000; RRID:AB_2080320), RPA (Abcam, ab2175, 1:500; RRID:AB_302873), pRPA-S4/8 (Bethyl, A-300-245, 1:1,000; RRID:AB_210547), actin (Cell Signaling Technology, #4970, 1:5,000; RRID:AB_2223172), and tubulin (Proteintech, 66031-1, 1:2,000; RRID:AB_2687491). Immunoblots were analyzed and quantified using Fiji (RRID:SCR_002285; Supplementary Data S1).

### RNA Sequencing and Analysis

RNA was isolated from cell lines using RNeasy Plus Mini Kit (Qiagen) and submitted to the Iowa Institute of Human Genetics Core Facility for analysis. Samples were barcoded, pooled, and sequenced on an Illumina NovaSeq 6000 (Illumina) to obtain a minimum of 30 million, paired-end, 100 bp reads per sample. FastQC was used to assess the quality of the sequencing reads. Reads were then mapped against the human reference genome (hg38) using the STAR aligner (STAR, RRID:SCR_004463). The raw counts were normalized and transformed using the rlog function and principal component analysis was performed to visualize sample clusters. No outlier samples were identified or removed from the analysis. The gene expression data were deposited in the Gene Expression Omnibus (GEO) Repository (RRID:SCR_005012) under the accession number GSE215881. The DESeq2 package (DESeq, RRID:SCR_000154) was used for the identification of differentially expressed (DE) genes. DE gene data were then analyzed for GSEA and transcription factor enrichment analysis (TFEA) using ShinyGO 0.76.1 and iDEP.96 (29, 30). The significance of enrichment was assessed using a hypergeometric test and FDR for multiple testing correction.

### Gemcitabine-resistant Cell Lines

Ewing sarcoma cell lines were cultured in progressively increasing concentrations of gemcitabine for approximately 3 months until the cells demonstrated >10-fold resistance to the drug.

### Clonogenic Assay

Clonogenic assays were performed as described previously (18). Cells were plated in 6-well plates in triplicate and then continuously treated with drug or vehicle for 10–14 days. Colonies were then stained with crystal violet and counted using an inverted Olympus CKX41 microscope.

### Cell-cycle Analysis

Cell-cycle analysis was performed in duplicate using the Click-iT EdU-488 kit for flow cytometry (Thermo Fisher Scientific). Cells were labeled with EdU for 2 hours and analysis was performed according to the manufacturer's instructions. Flow cytometry was performed on a Becton Dickinson LSR II instrument.

### siRNA Transfection

Cells (1.5–3 × 10^5^) were plated one day prior to transfection in 6-well plates. Cells were transfected with siRNA using Lipofectamine RNAiMax (Thermo Fisher Scientific) according to the manufacturer's instructions. siSLFN11 was a SMARTpool ON-TARGETplus reagent (GE Dharmacon) and siControl was obtained from Cell Signaling Technology (#6568).

### Doxycycline-inducible Expression of c-Jun and c-Fos

The full-length c-Jun and c-Fos cDNAs, separated by a T2A element, were synthesized and inserted into a pLV-TRE lentiviral vector downstream of the TRE3G doxycycline-inducible promoter (VectorBuilder). The pLVX-EF1a-Tet3G vector (Takara Bio) was used to express the Tet-On 3G transactivator protein from the human EF1 alpha promoter. Lentivirus was prepared as described above. Ewing sarcoma cells were sequentially infected and selected with geneticin 500 μg/mL (pLVX-EF1a-Tet3G) and puromycin 1 μg/mL (pLV-TRE3G-c-Jun-T2A-c-Fos).

### AP-1 Reporter Assay

A lentiviral, AP-1 luciferase reporter plasmid was obtained from System Biosciences (pGreenFire 2.0 AP-1 Reporter, TR452PA-P). Lentivirus was prepared as described above and cells were selected in puromycin 1 μg/mL. Luciferase activity was quantified using luciferin (Sigma) and a FLUOstar Omega microplate reader (BMG Labtech).

### qRT-PCR

Total RNA was extracted from cells using a RNeasy kit (Qiagen) following the manufacturer's instructions. A total of 1 μg of total RNA was reverse transcribed into first-strand cDNA using random hexamer primers and the SuperScript III Reverse Transcriptase (Thermo Fisher Scientific). qRT-PCR was performed on the ViiA 7 Real-Time PCR System (Life Technologies) using SYBR Select Master Mix (Thermo Fisher Scientific). Reactions were performed in triplicate and gene expression was normalized to GAPDH. The PCR primer sequences for RRM1, MYC, and GAPDH were 5′-GCCAGGATCGCTGTCTCTAAC-3′, 5′-GGCTCCTGGCAAAAGGTCA-3′, and 5′-CTGGGCTACACTGAGCACC-3′, respectively.

### Xenograft

The Institutional Animal Care and Usage Committee at the University of Iowa (Iowa City, IA) approved the animal experiments, and the studies were conducted in adherence with the NIH Guide for the Care and Use of Laboratory Animals. The tumor samples used in this study were previously collected for an earlier study (28). Briefly, approximately 1.0 × 10^6^ EW8 or TC71 cells were mixed with 30% matrigel and injected subcutaneously into the flanks of 6-week-old, female NCr mice. After tumors were palpable (∼100–200 mm^3^), the mice were treated with vehicle or gemcitabine (150 mg/kg, intraperitoneal, day 1). On day 2, the mice were sacrificed and the tumors were collected for analysis by immunoblotting.

### Statistical Analysis

Two-tailed Student *t* test was used to calculate *P* values for the comparison of two groups. Analyses for more than two groups were conducted with a one-way ANOVA followed by Dunnett multiple comparisons test. Statistical analyses were conducted using GraphPad Prism 9.

### Data Availability

The data generated in this study are available within the article and its Supplementary Data. The gene expression data were deposited in the GEO Repository under the accession number GSE215881.

## Results

### Conditional Knockout of RRM1 Causes Cell-cycle Arrest, DNA Damage, and Apoptosis in Ewing Sarcoma Cells

In previous work, we used a stem cell model and gene expression–based screening approach to identify that Ewing sarcoma cells are uniquely sensitive to RNR inhibitors, including gemcitabine (19, 20, 28). Analysis of two large-scale drug screening data sets (Genomics of Drug Sensitivity in Cancer, Wellcome Sanger Institute, and Massachusetts General Hospital) also identified a highly significant association between the pathognomonic genomic marker in Ewing sarcoma tumors, EWS-FLI, and sensitivity to gemcitabine (Supplementary Fig. S1A and S1B; ref. 26). However, as discussed above, the polypharmacology of gemcitabine and other RNR inhibitors has complicated the identification of the precise mechanism of action of these drugs in Ewing sarcoma and other tumors.

**FIGURE 1:**
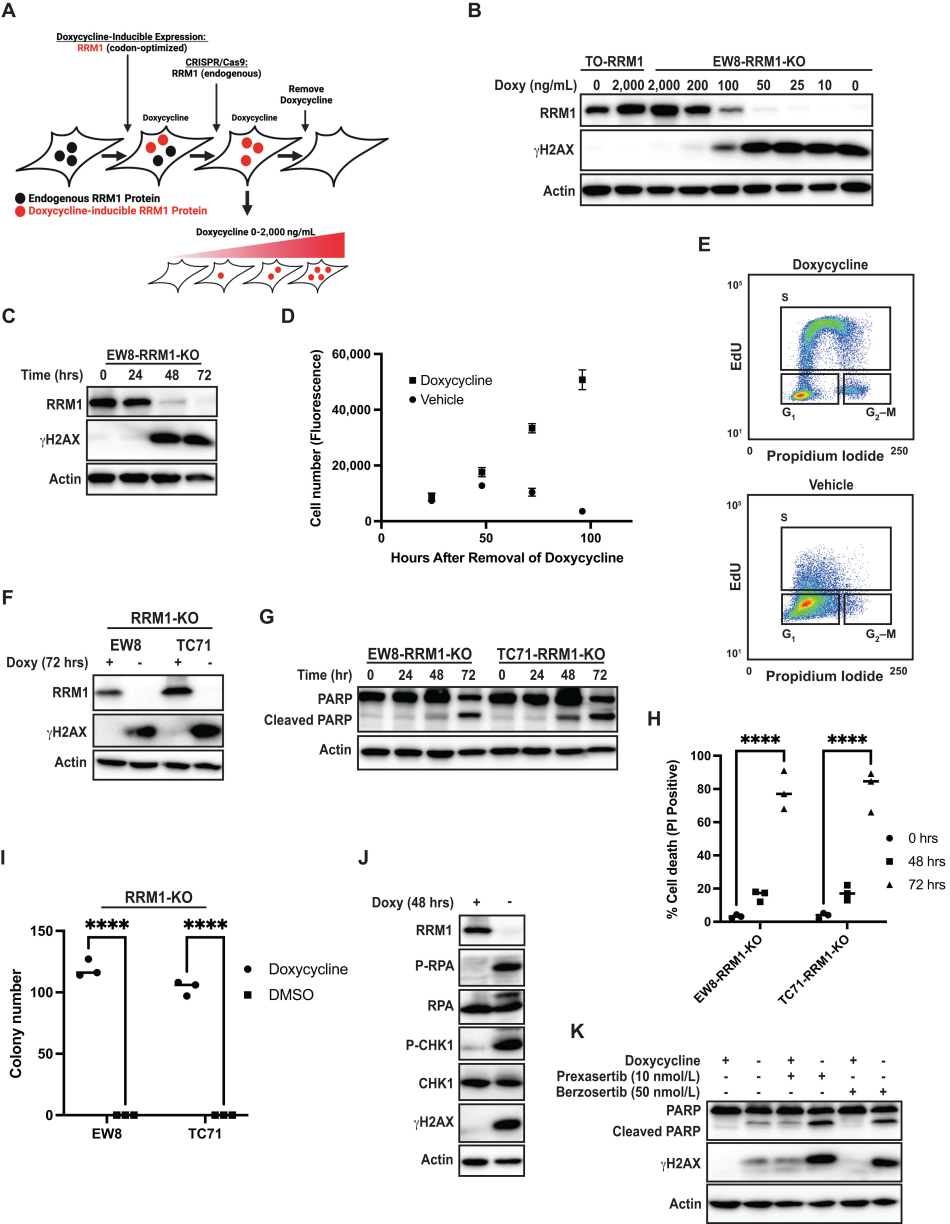
Conditional knockout of RRM1 causes cell-cycle arrest, DNA damage, and apoptosis in Ewing sarcoma cells. **A,** Schematic illustrating the approach used to knockout the endogenous RRM1 gene in Ewing sarcoma cells that express an exogenous and doxycycline-inducible RRM1 transgene that is codon optimized and resistant to targeting by CRISPR/Cas9. **B,** EW8-TO-RRM1 and EW8-RRM1-KO cells were treated with different concentrations of doxycycline for 72 hours and then cellular lysates were collected for immunoblotting. **C,** Doxycycline was removed from the EW8-RRM1-KO cells for 24–72 hours and cellular lysates were collected for immunoblotting. **D,** Growth assay for EW8-RRM1-KO cells with and without doxycycline. Cell viability was assessed at different timepoints using the AlamarBlue Fluorescence Assay. The results are representative of two independent experiments. Error bars represent mean ± SD of three technical replicates. **E,** Representative cell-cycle analysis of EW8-RRM1-KO cells growing with or without of doxycycline for 48 hours. **F,** Doxycycline was removed from the EW8-RRM1-KO and TC71-RRM1-KO cell lines for 72 hours and then cellular lysates were collected for immunoblotting. **G,** Doxycycline was removed from the EW8-RRM1-KO and TC71-RRM1-KO cell lines and lysates were collected at different time points. **H,** Doxycycline was removed from the EW8-RRM1-KO and TC71-RRM1-KO cell lines for different amounts of time and then dead cells were labeled with propidium iodide and quantified using flow cytometry. **I,** Colony formation assay for the EW8-RRM1-KO and TC71-RRM1-KO cell lines in the presence or absence of doxycycline for 10–12 days. **J,** Doxycycline was removed from the EW8-RRM1-KO cell line for 48 hours and then cellular lysates were collected for immunoblotting for markers of DNA replication stress. **K,** EW8-RRM1-KO cells were grown with or without doxycycline for 24 hours, at which point the cells were treated with vehicle (DMSO), prexasertib (CHK1 inhibitor), or berzosertib (ATR inhibitor) for an additional 24 hours. Cell lysates were then collected for immunoblotting. *P* values were calculated using a two-tailed Student *t* test. ****, *P* < 0.0001.

We used CRISPR/Cas9 to knockout RRM1, which is the catalytic subunit of RNR and the target of gemcitabine, in Ewing sarcoma cells. We chose to focus on the RRM1 subunit of RNR rather than the RRM2 subunit because the RRM2B protein, which shares 83% sequence homology with RRM2, can dimerize with RRM1 and form an active RNR holoenzyme (1). In addition, the level of the RRM2 subunit varies during the cell cycle, whereas RRM1 is expressed at a constant level and is more amenable to rescue using a doxycycline-inducible expression approach (31, 32). However, before knocking out the RRM1 gene, we validated that the endogenous RRM1 protein is expressed at a stable level throughout the cell cycle in Ewing sarcoma cells by labeling EW8 cells with a fluorescent, cell-cycle reporter (PIP-FUCCI) and using flow cytometry to isolate cells in G_1_, S, and G_2_–M phases of the cell cycle (27). Supplementary Figure S1C shows that the level of the RRM1 protein does not vary between the different phases of the cell cycle, in contrast to the level of the RRM2 protein which peaks during S phase. RRM1 is an essential gene so we used CRISPR/Cas9 to knockout the endogenous RRM1 gene in EW8 cells that express an exogenous, doxycycline-inducible, and Cas9-resistant RRM1 transgene (TO-RRM1; Fig. 1A; Supplementary Fig. S1D). Figure 1B and C demonstrate that removal of doxycycline from the EW8-RRM1-KO cells reduced the level of the RRM1 protein in a dose- and time-dependent fashion, which correlated with the upregulation of a marker of DNA damage (γH2AX). Notably, the level of the RRM1 protein was undetectable by 48–72 hours after removal of doxycycline (Fig. 1C) and this timepoint coincided with a decrease in cell growth (Fig. 1D) and cell-cycle arrest in late G_1_/early S phase (Fig. 1E).

Next, we generated a second Ewing sarcoma cell line (TC71) with conditional knockout of RRM1 (Fig. 1F). Live cell imaging revealed that removal of doxycycline in both cell lines resulted in morphologic changes indicative of apoptosis and cell death at 72 hours (Supplementary Fig. S1E). Increased cleavage of PARP, a marker of apoptosis, and cellular uptake of propidium iodide, a marker of cell death, were also observed 72 hours after removal of doxycycline (Fig. 1G and H). Furthermore, removal of doxycycline eliminated colony formation in both cell lines (Fig. 1I). Inhibition of RNR and induction of DNA replication stress in Ewing sarcoma tumors generates single-strand DNA (ssDNA) and activates the replication stress response pathway (20, 33, 34). Figure 1J shows that removal of doxycycline and loss of RRM1 resulted in phosphorylation of replication protein A (RPA) and checkpoint kinase 1 (CHK1), which are critical mediators of the cellular response to replication stress (35). Furthermore, consistent with published work demonstrating synergy between RNR and ATR-CHK1 inhibitors, the removal of doxycycline also increased phosphorylation of CHK1 and enhanced the sensitivity of the cell lines to inhibitors of ATR and CHK1 (Fig. 1K; Supplementary Fig. S2; refs. 20, 36).

### Loss of RNR Activity in Ewing Sarcoma Cells Increases Expression of Members of the AP-1 Transcription Factor Complex

**FIGURE 2:**
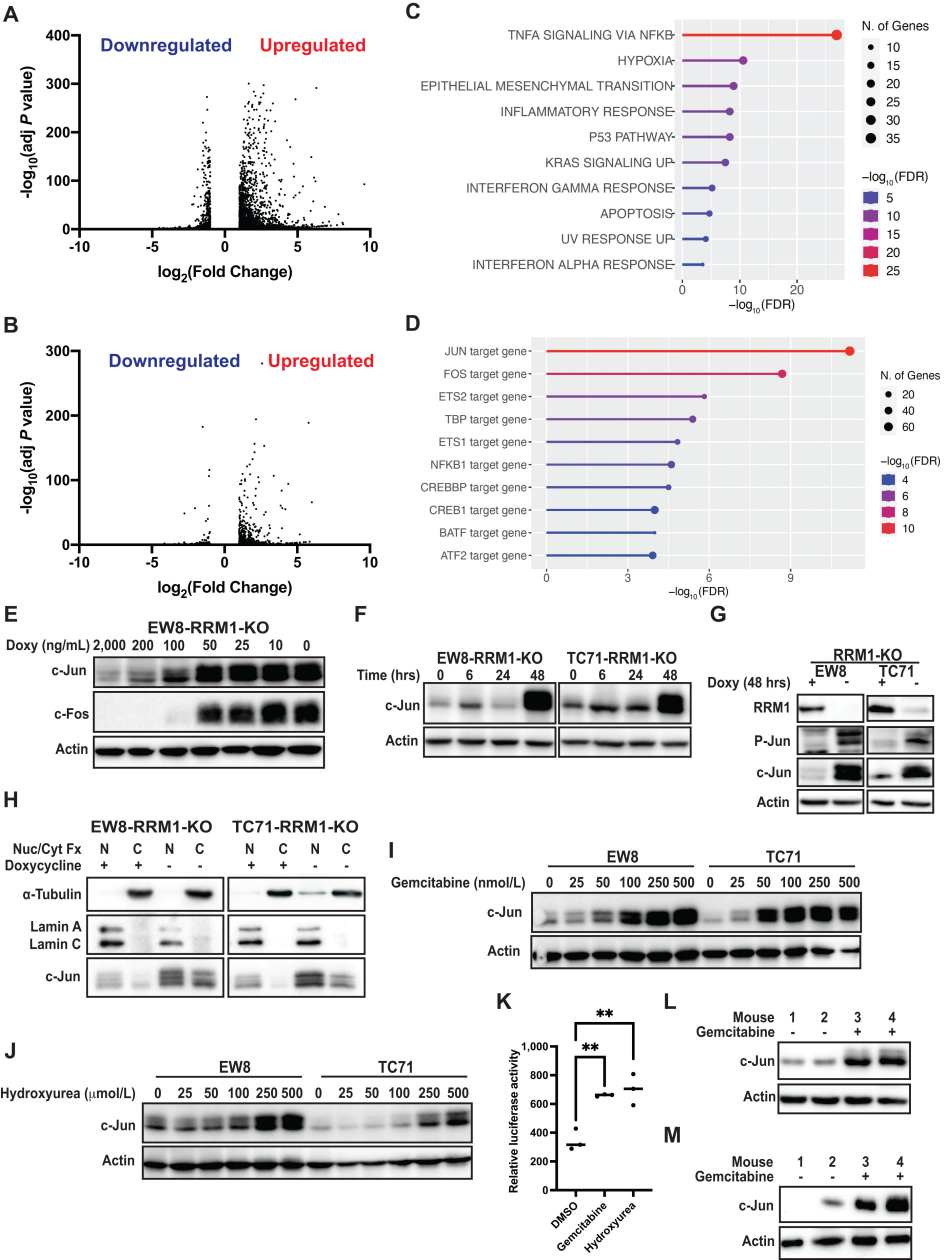
Loss of RRM1 activates AP-1 signaling in Ewing sarcoma cells. Volcano plots of DE genes (fold >2, adjusted *P* value <0.05) in the EW8-RRM1-KO (**A**) and TC71-RRM1-KO (**B**) cell lines in the presence and absence (48 hours) of doxycycline. **C,** Gene sets (biological processes) enriched in the 412 overlap genes that are upregulated in both the EW8-RRM1-KO and TC71-RRM1-KO cell lines in the absence of doxycycline. **D,** Results of TFEA performed with the overlap genes that are upregulated in both the EW8-RRM1-KO and TC71-RRM1-KO cell lines in the absence of doxycycline. **E,** EW8-RRM1-KO cells were treated with different concentrations of doxycycline for 48 hours and then cellular lysates were collected for immunoblotting. **F,** Doxycycline was removed from the EW8-RRM1-KO and TC71-RRM1-KO cell lines and lysates were collected at different timepoints. **G,** Doxycycline was removed from the EW8-RRM1-KO and TC71-RRM1-KO cells for 48 hours. Cell lysates were then collected for immunoblotting for total and phosphorylated (Ser73) c-Jun. **H,** EW8-RRM1-KO and TC71-RRM1-KO cells were grown with or without doxycycline for 48 hours. Subcellular fractionation was then performed and lysates were immunoblotted for c-Jun and markers of the nuclear (α-Tubulin) and cytoplasmic (Lamin A/C) fractions. The parental, unmodified EW8 and TC71 cells were treated with a range of gemcitabine (**I**) or hydroxyurea doses (**J**) for 24 hours and then cell lysates were collected for immunoblotting. **K,** EW8 cells labeled with an AP-1 (luciferase) reporter were used to quantify AP-1 activity when cell lines were treated with gemcitabine or hydroxyurea for 24 hours. TC71 (**L**) and EW8 (**M**) cells were engrafted in nude (NCr) mice. After tumors were palpable, the mice were treated with vehicle or gemcitabine (150 mg/kg, intraperitoneal, day 1). On day 2, the mice were sacrificed and the tumors were collected for analysis by immunoblotting. *P* values were calculated using a two-tailed Student *t* test or a one-way ANOVA followed by Dunnett multiple comparisons test. **, *P* < 0.01.

Next, we performed RNA sequencing (RNA-seq) analysis with the EW8 and TC71 conditional knockout cell lines to identify genes and pathways upregulated and downregulated by the loss of RRM1 activity. Removal of doxycycline from both the EW8-RRM1-KO and TC71-RRM1-KO cell lines resulted in more upregulated genes than downregulated genes (Fig. 2A and B). There was significant overlap between the DE genes in the EW8-RRM1-KO and TC71-RRM1-KO cell lines, although more genes were dysregulated in the EW8-RRM1-KO cell line than the TC71-RRM1-KO cell line (Supplementary Fig. S3A). There were 412 overlap genes upregulated in both cell lines and GSEA using these genes identified significant (FDR *P* value <0.05) enrichment for TNFα signaling, hypoxia, apoptosis, differentiation, and epithelial–mesenchymal transition gene sets (Fig. 2C; Supplementary Fig. S3B; ref. 29). Similar results were obtained using the RNA-seq data for the individual cell lines (Supplementary Table S1). We then analyzed the upregulated genes with TFEA and identified that the top transcription factors with significant enrichment for regulation of this set of genes were c-Jun and c-Fos, which are AP-1 transcription factor family proteins (Fig. 2D). The AP-1 transcription factor is a dimeric complex that is composed of members of the JUN, FOS, ATF (activating transcription factor), and MAF (musculoaponeurotic fibrosarcoma) protein families, and multiple members of this family in addition to c-Jun and c-Fos were upregulated by loss of RRM1 (Table 1; Supplementary Fig. S3C and S3D; refs. 23, 24). Finally, although more genes were upregulated than downregulated, GSEA also identified that the downregulated genes in both cell lines were enriched for c-Myc target genes (Supplementary Table S1).

**TABLE 1:** Increase in mRNA expression levels of AP-1 family members in RRM1-KO cell lines after removal of doxycycline and loss of the RRM1 protein

	log_2_ Fold Increase	
Gene	EW8-RRM1-KO	TC71-RRM1-KO
*JUN*	6.5	4.4
*JUNB*	2.5	1.7
*FOS*	6.3	5.8
*FOSB*	5.1	3.9
*FOSL1*	3.6	1.6
*ATF3*	4.9	3.4
*MAFF*	3.9	1.9
*BATF2*	3.1	1.0

Immunoblotting for c-Jun and c-Fos identified upregulation of both proteins at 48 hours after removal of doxycycline (Fig. 2E and F). In addition, removal of doxycycline increased phosphorylation of c-Jun at Ser73, which enhances c-Jun transcriptional activity (Fig. 2G), and nuclear localization of c-Jun (Fig. 2H; refs. 37, 38). Then, to confirm that the results of our genetic knockout system are applicable to clinical RNR inhibitors, we treated the parental, unmodified Ewing sarcoma cell lines with gemcitabine or hydroxyurea. Figure 2I and J show that drugs that target either the RRM1 (gemcitabine) or RRM2 (hydroxyurea) subunit of RNR increase the expression level of c-Jun. The expression of c-Jun, which is an immediate early gene (IEG), can be rapidly induced in response to stress and Supplementary Fig. S4A demonstrates robust upregulation of c-Jun in response to 6 hours of treatment with gemcitabine. We also used an AP-1 (luciferase) reporter to quantify AP-1 activity when cell lines were treated with RNR inhibitors. Figure 2K shows that both gemcitabine and hydroxyurea significantly increased luciferase activity in Ewing sarcoma cells. DNA replication stress has been reported to upregulate AP-1 in other cancer types, but treatment of nontransformed cells (RPE-tert and BJ-tert) with gemcitabine did not increase expression of c-Jun or c-Fos (Supplementary Fig. S4B; refs. 39, 40).

We then generated gemcitabine-resistant Ewing sarcoma cells by culturing cells in progressively increasing concentrations of the drug (Supplementary Fig. S5A). Gemcitabine-resistant cells, in contrast to the gemcitabine-sensitive cells, did not upregulate c-Jun expression in response to treatment with gemcitabine (Supplementary Fig. S5B). In addition, treatment of the EW8-RRM1-KO cells for 5 days with low-dose doxycycline (100 ng/mL), which partially reduces the level of RRM1 (Fig. 1B) and slows cell growth without complete cell-cycle arrest, also increased expression of c-Jun (Supplementary Fig. S5C and S5D). Finally, EW8 and TC71 Ewing sarcoma cells were engrafted in nude (NCr) mice. After tumors were palpable, the mice were treated with vehicle or gemcitabine. The mice were sacrificed 24 hours after drug treatment and the tumors were collected for analysis by immunoblotting. Figure 2L and M demonstrate that gemcitabine treatment upregulates c-Jun *in vivo* in xenograft tumors.

### Expression of c-Jun and c-Fos in Ewing Sarcoma Cells Downregulates the Expression of c-Myc and Inhibits Proliferation

**FIGURE 3:**
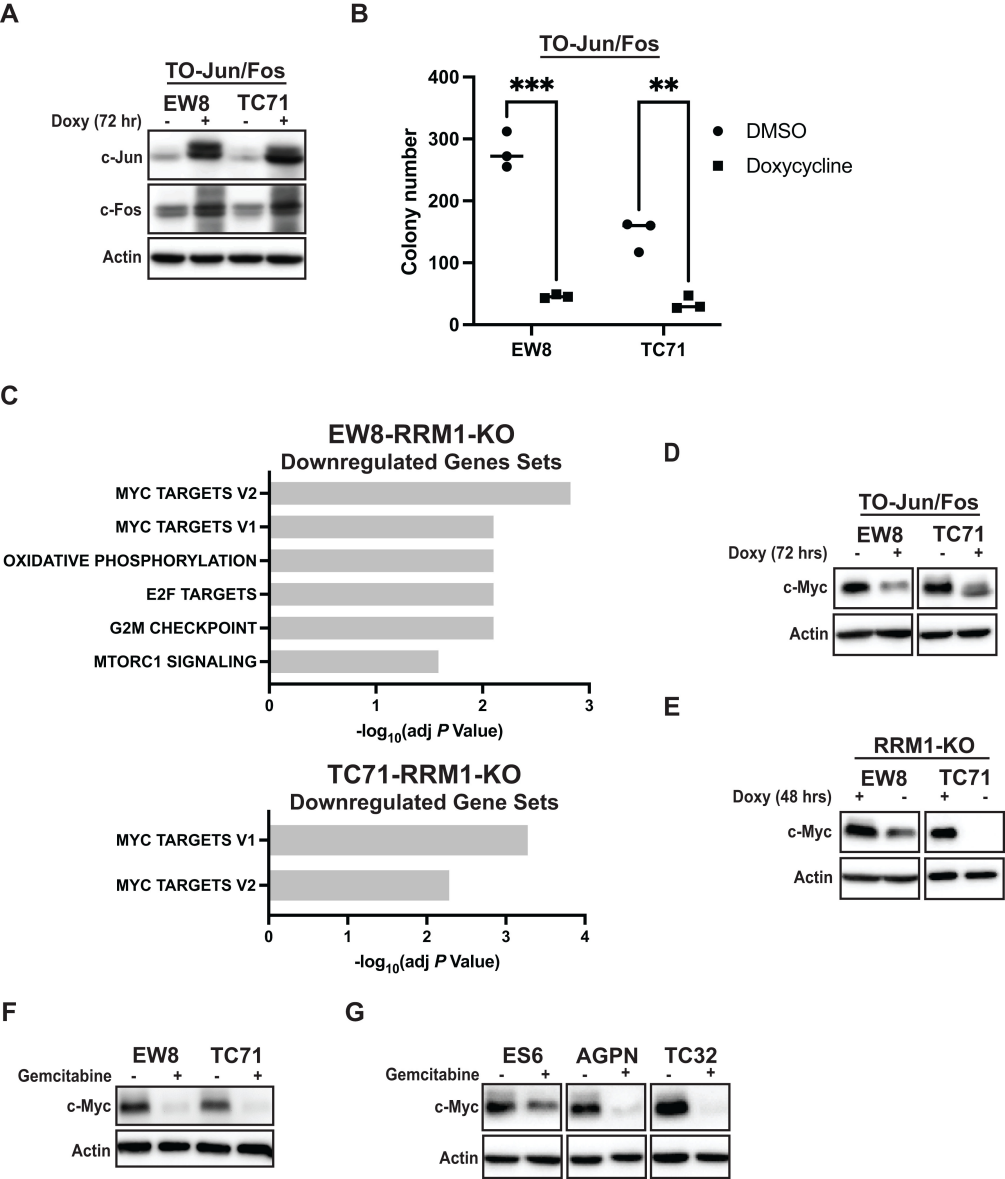
Expression of c-Jun and c-Fos in Ewing sarcoma cells inhibits cell growth and downregulates c-Myc. **A,** EW8 and TC71 cell lines expressing doxycycline-inducible c-Jun and c-Fos were grown with or without doxycycline for 24 hours. Cell lysates were collected for immunoblotting. **B,** Colony formation assay for the TO-Jun/Fos cell lines grown with or without doxycycline for 10–12 days. **C,** Results of GSEA for gene sets downregulated in the EW8-RRM1-KO and TC71-RRM1-KO cell lines after removal of doxycycline for 48 hours. **D,** The TO-EW8-Fos/Jun and TO-TC71-Fos/Jun cell lines were grown with or without doxycycline for 72 hours. Cell lysates were then collected for immunoblotting. **E,** The EW8-RRM1-KO and TC71-RRM1-KO cell lines were grown with or without doxycycline for 48 hours. Cell lysates were then collected for immunoblotting. **F** and **G,** Ewing sarcoma cell lines were treated with gemcitabine (100 nmol/L) for 24 hours and then cell lysates were collected for immunoblotting. *P* values were calculated using a two-tailed Student *t* test. **, *P* < 0.01; ***, *P* < 0.001.

AP-1 activity is modulated through its dimer composition and the function of AP-1 is highly cell and context dependent so we used a doxycycline-inducible, lentiviral system to simultaneously overexpress both c-Jun and c-Fos in the parental EW8 and TC71 cell lines (Fig. 3A; refs. 23, 24). Expression of c-Jun and c-Fos in these cell lines significantly inhibited cell growth in colony formation assays (Fig. 3B). AP-1 is reported to regulate c-Myc, which promotes tumorigenesis in Ewing sarcoma tumors, and GSEA with the RRM1 knockout cells identified that loss of RRM1 activity results in the downregulation of c-Myc target genes (Fig. 3C; Supplementary Fig. S6A and S6B; Supplementary Table S1; refs. 41–47). Consequently, we performed immunoblotting for c-Myc and confirmed that both overexpression of Jun/Fos and loss of RRM1 activity downregulate the level of the c-Myc protein (Fig. 3D and E; refs. 42, 43, 45–47). Treatment of the parental EW8 and TC71 cells, as well as additional Ewing sarcoma cell lines, with the RRM1 inhibitor gemcitabine also reduced the level of the c-Myc oncoprotein (Fig. 3F and G). qRT-PCR analysis with the TO-Jun/Fos cell lines did not identify a significant change in the level of c-Myc mRNA, which suggests that AP-1 regulates c-MYC levels via a nontranscriptional mechanism (Supplementary Fig. S6C). Similar results regarding c-MYC mRNA levels were also observed in the RNA-seq data, as well as qRT-PCR, with the RRM1-KO cell lines (Supplementary Fig. S6D). The EWS-FLI oncogene is known to upregulate c-Myc expression, but the level of the EWS-FLI protein was not decreased by expression of c-Jun and c-Fos (Supplementary Fig. S6E).

### SLFN11 Contributes to the Toxicity of RNR Inhibition and the Upregulation of AP-1

**FIGURE 4:**
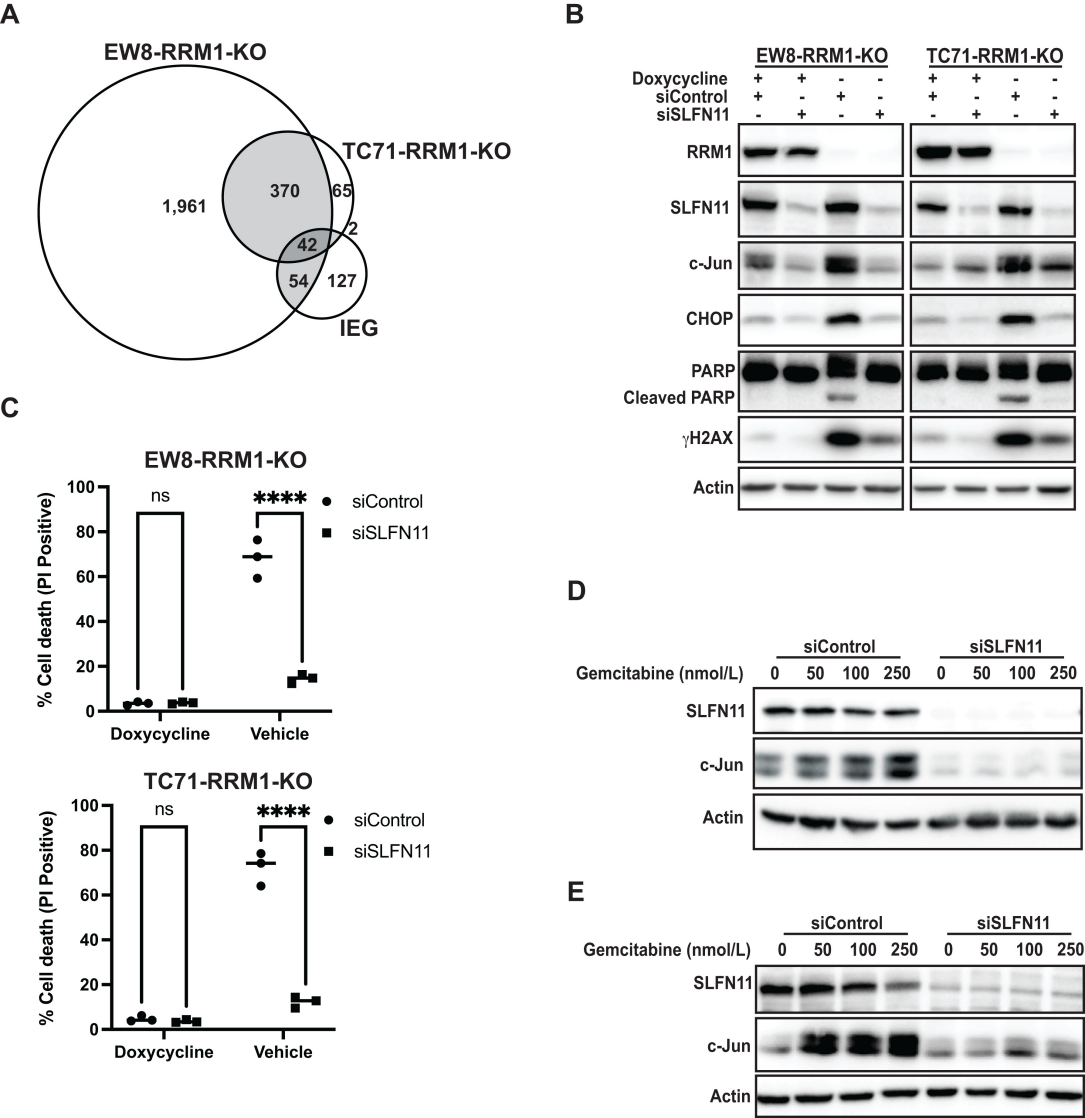
SLFN11 contributes to the toxicity of RNR inhibition and the upregulation of AP-1. **A,** Venn diagram demonstrating the overlap between genes upregulated in the EW8-RRM1-KO and TC71-RRM1-KO cells grown in the absence of doxycycline and IEGs. **B,** EW8-RRM1-KO and TC71-RRM1-KO cells were grown with or without doxycycline for 24 hours, at which point the cells were transfected with control or SLFN11 siRNA and grown for an additional 24 hours. Cell lysates were then collected for immunoblotting. **C,** EW8-RRM1-KO and TC71-RRM1-KO cells were grown with or without doxycycline for 24 hours, at which point the cells were transfected with control or SLFN11 siRNA and grown for an additional 48 hours. Dead cells were then labeled with propidium iodide and quantified using flow cytometry. EW8 (**D**) and TC71 (**E**) cell lines were treated with control or SLFN11 siRNA for 24 hours. Gemcitabine was then added for an additional 24 hours before collecting cellular lysates. *P* values were calculated using a two-tailed Student *t* test. ****, *P* < 0.0001.

Schlafen family member 11 (SLFN11) is a transcriptional target of EWS-FLI1 and sensitizes Ewing sarcoma cells, as well as other cancer types, to a variety of DNA-damaging agents, including gemcitabine and additional RNR inhibitors (19, 21, 25, 39, 48, 49). Notably, SLFN11 binds to ssDNA at stalled DNA replication forks and induces, via alterations in chromatin accessibility, the expression of IEGs, which include the AP-1 family members c-Jun and c-Fos (50). Figure 4A shows the 42 IEGs are upregulated in both the EW8-RRM1-KO and TC71-RRM1-KO cell lines after removal of doxycycline (51). Next, to determine whether SLFN11 regulates AP-1 expression, we used siRNA to knockdown SLFN11 in the RRM1-KO cell lines. Figure 4B shows that the knockdown of SLN11 blocked the upregulation of c-Jun, as well as markers of DNA damage (γH2AX) and apoptosis (cleaved PARP), in the setting of loss of RRM1. Knockdown of SLN11 also significantly decreased the toxicity caused by loss of RRM1, as assessed using propidium iodide to quantify dead cells (Fig. 4C). Similarly, siRNA-mediated knockdown of SLN11 in the parental, unmodified EW8 and TC71 cells also abrogated the upregulation of c-Jun in response to treatment with gemcitabine (Fig. 4D and E).

### HDAC Inhibitors Decrease the Level of the RRM1 Protein and Increase Expression of c-Jun in Ewing Sarcoma Cells

**FIGURE 5:**
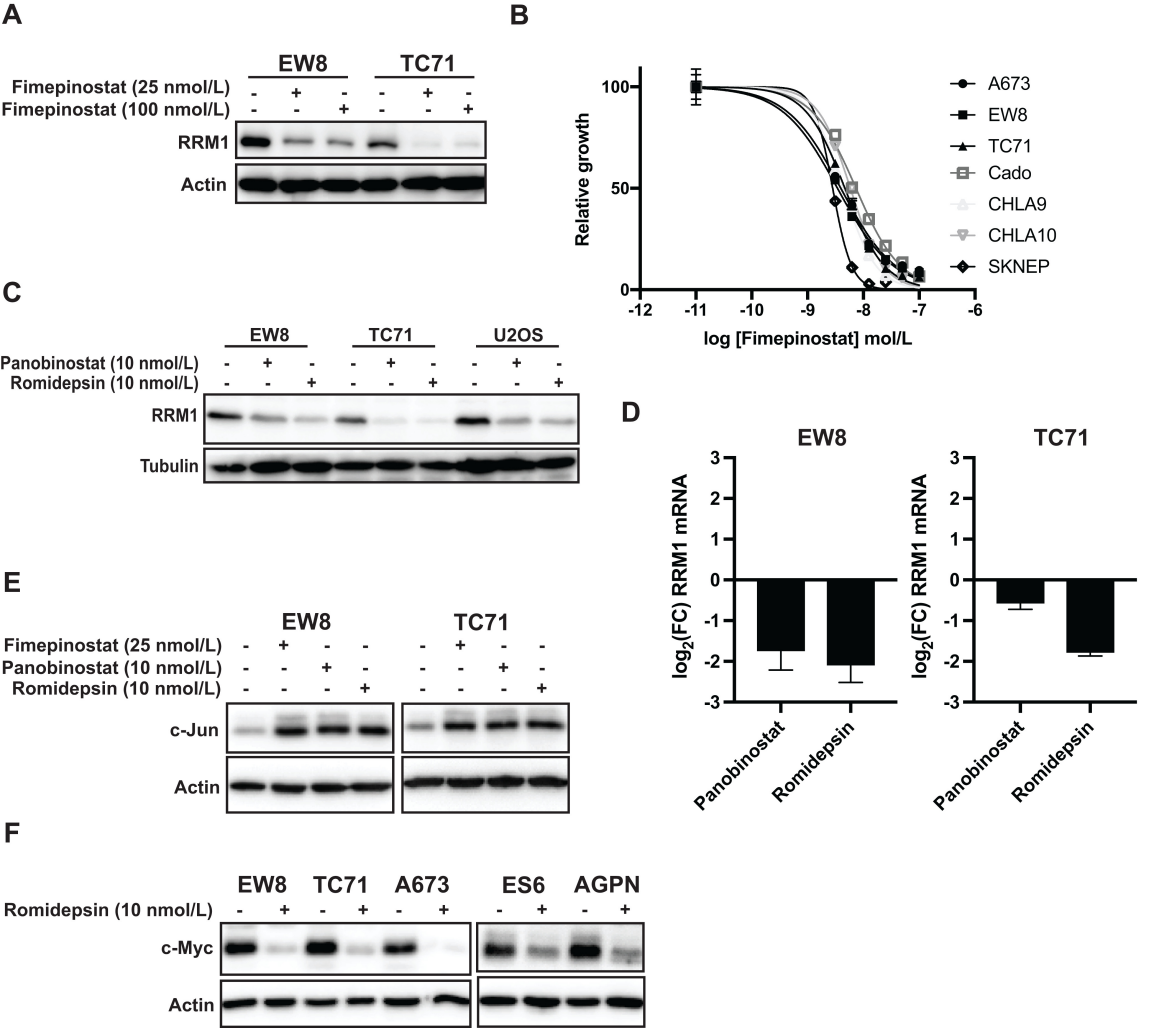
HDAC inhibitors decrease the level of the RRM1 protein and increase expression of c-Jun in Ewing sarcoma cells. **A,** EW8 and TC71 cell lines were treated with the dual PI3K-HDAC inhibitor fimepinostat for 24 hours and then cell lysates were collected for immunoblotting. **B,** Dose–response curves for Ewing sarcoma cell lines treated with different concentrations of fimepinostat for 72 hours. Cell viability was assessed using the AlamarBlue Fluorescence Assay. The results are representative of two independent experiments. Error bars represent mean ± SD of three technical replicates. **C,** Ewing sarcoma (EW8 and TC71) and osteosarcoma (U2OS) cell lines were treated with the HDAC inhibitors panobinostat or romidepsin for 24 hours and then cell lysate was collected for immunoblotting. **D,** log_2_ fold change (FC) in RRM1 mRNA in EW8 and TC71 cells treated with panobinostat or romidepsin for 24 hours. The results are representative of two independent experiments. Error bars represent the mean ± SD of three technical replicates. **E,** EW8 and TC71 cells were treated with panobinostat, romidepsin, or fimepinostat for 24 hours and then cell lysates were collected for immunoblotting. **F,** Ewing sarcoma cell lines were treated with romidepsin for 24 hours and then cell lysates were collected for immunoblotting.

We identified that a reduction in the level of the RRM1 protein causes an increase in AP-1 signaling, and similar results were obtained using drugs that inhibit the activity of RNR. However, inhibition of an enzyme or protein is not always functionally equivalent to a reduction in the level of that protein (52). Consequently, we wanted to test the hypothesis that drugs that decrease the level of the RRM1 protein will also upregulate AP-1. We previously identified, in unpublished work, that HDAC inhibitors decrease the level of the RRM1 protein in Ewing sarcoma and other cell types. Figure 5A and B demonstrate that the treatment of Ewing sarcoma cell lines with the pan-HDAC inhibitor fimepinostat reduced the level of the RRM1 protein and inhibited the growth of Ewing sarcoma cell lines in a dose–response growth assay (53). Fimepinostat is a dual inhibitor of HDAC and PI3K so we then tested two additional HDAC inhibitors that are currently used in the clinic, romidepsin and panobinostat (54). Figure 5C shows that these additional HDAC inhibitors also decreased the level of the RRM1 protein in Ewing sarcoma cells, as well as an osteosarcoma cell line (U2OS). In addition, qRT-PCR analysis identified that romidepsin and panobinostat both reduced the level of RRM1 mRNA in the EW8 and TC71 cell lines (Fig. 5D). Finally, similar to the results obtained with the RRM1-KO and TO-Jun/Fos cell lines, treatment of Ewing sarcoma cells with these HDAC inhibitors also increased c-Jun expression and decreased c-Myc expression (Fig. 5E and F).

## Discussion

**FIGURE 6:**
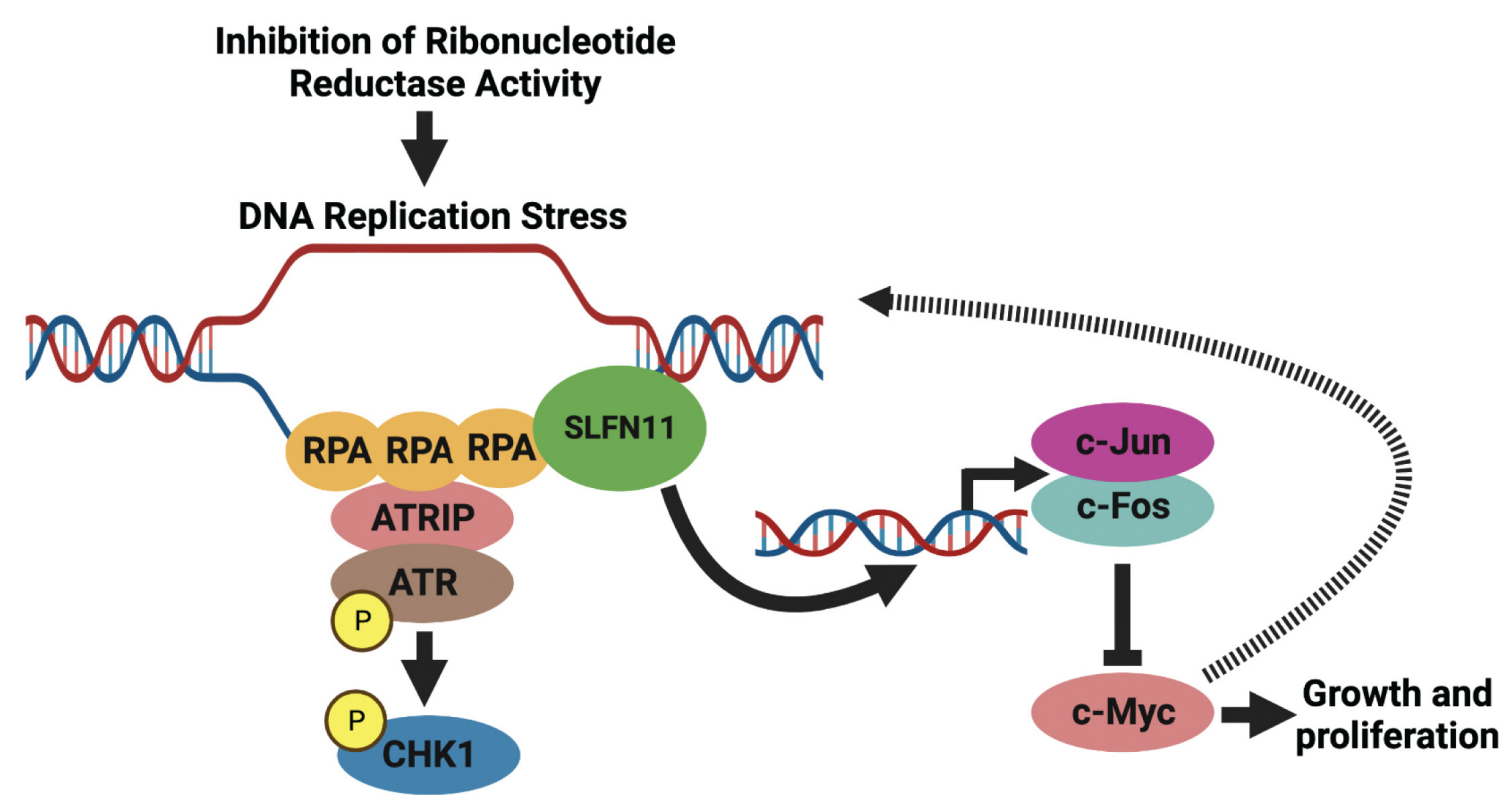
Proposed mechanism for the activation of the ATR-CHK1 and AP-1 signaling pathways in response to inhibition of RNR activity in Ewing sarcoma cells. Inhibition or loss of RNR activity in Ewing sarcoma cells causes DNA replication stress, generates ssDNA, and activates RPA and ATR-CHK1 signaling. SLFN11 interacts with RPA at the stalled replication forks, blocks replication restart, and upregulates the expression of IEGs c-Jun and c-Fos via effects on the chromatin. c-Jun and c-Fos then inhibit the growth of Ewing sarcoma cells, in part, by decreasing the level of the c-Myc oncogene, which promotes proliferation and tumorigenesis in Ewing sarcoma tumors. In addition, c-Myc multimers are reported to stabilize stalled DNA replication forks, which suggests that the downregulation of c-Myc caused by loss of RRM1 could also enhance DNA damage via fork destabilization.

Inhibitors of RNR are widely used in clinical oncology, but the precise mechanism of how these drugs induce toxicity in cancer cells is poorly understood due to polypharmacology and off-target effects (1, 13, 15–17). For example, gemcitabine inhibits the RRM1 subunit of RNR and depletes deoxyribonucleotides, but this nucleoside analog is also incorporated into DNA where it blocks chain elongation and causes DNA damage (10). In this work, we identified that loss of RNR activity, via a conditional knockout approach targeting the RRM1 subunit of RNR, is sufficient to cause cell-cycle arrest, DNA damage, and apoptosis in Ewing sarcoma cells. However, we also found that the loss of RRM1 in Ewing sarcoma cells upregulates the expression of multiple AP-1 transcription factors, including the canonical family members c-Jun and c-Fos, and downregulates the expression of c-Myc (Fig. 6; refs. 23, 24). Furthermore, we identified that the expression of c-Jun and c-Fos in Ewing sarcoma cells is sufficient to inhibit cell growth and reduce the expression of the c-Myc oncogene (Fig. 3).

The AP-1 transcription factor, which can serve as both an oncogene and tumor suppressor, is activated by diverse cellular stressors and functions as a homodimeric and heterodimeric protein complex composed of different combinations of members from the JUN, FOS, ATF, and MAF protein families (23, 24, 55–57). AP-1 has many downstream targets and is known to regulate a diverse set of genes related to oncogenesis, apoptosis, and differentiation (23, 57, 58). In this work, we identified that AP-1, in the setting of loss of RRM1 activity, downregulates the level of the c-Myc oncogene, which promotes tumorigenesis in Ewing sarcoma tumors via multiple pathways (42, 43, 46, 47). In addition, recent work identified that c-Myc multimers can shield stalled replication forks from damage and limit double-strand break formation in the setting of replication stress, which suggests that the downregulation of c-Myc caused by loss of RRM1 could also function in a feedback loop and enhance DNA damage via fork destabilization (59). However, whether c-Myc multimers stabilize stalled replication forks in Ewing sarcoma cells is unknown and will be investigated in future work. The mechanism of how AP-1 regulates c-Myc levels in Ewing sarcoma cells is also unknown. Although AP-1 binding sites were previously identified in the c-Myc promoter, our RNA-seq and qRT-PCR data did not identify a decrease in c-Myc mRNA, which suggests that AP-1 may regulate c-Myc levels via an indirect and nontranscriptional mechanism (41, 60). In addition, although we chose to focus our investigation on c-Jun and c-Fos because these transcription factors are the canonical and most well-described members of the AP-1 family, the gene expression data identified the upregulation of additional members of the AP-1 family (Table 1). Consequently, differentiating the individual roles and contributions of these AP-1 family members, as well as identifying additional downstream targets of the complex, will be areas of future investigation.

Upregulation of AP-1 in Ewing sarcoma cells is dependent on SLFN11, which is a direct transcriptional target of the EWS-FLI1 oncoprotein and highly expressed in Ewing sarcoma tumors (25, 48–50). In addition, Tang and colleagues showed that patients with Ewing sarcoma with higher SLFN11 expression exhibited better prognosis than those with lower SLFN11 expression (49). SLFN11 is known to activate stress response pathways in cells and induces, via alterations in chromatin accessibility, the expression of IEGs, including the AP-1 family members c-Jun and c-Fos (50). For example, Murai and colleagues showed that DNA replication stress induces the SLFN11-dependent upregulation of c-Jun and c-Fos expression in leukemia and prostate cancer cell lines (50). Furthermore, the camptothecin-induced upregulation of multiple AP-1 transcription factors is significantly correlated with the basal *SLFN11* expression levels for 55 cell lines (NCI-60 panel) representing multiple cancer types. However, in addition to upregulating the expression of IEGs, SLFN11 also blocks the restart of DNA replication at stalled DNA replication forks, which leads to fork collapse and DNA damage (39). Dissecting the relative impact on cell viability of these different functions of SLFN11 will be the focus of future investigation.

SLFN11 is highly expressed in additional types of pediatric sarcomas, including osteosarcoma, embryonal rhabdomyosarcoma, and desmoplastic small round cell tumor (61). This suggests that the AP-1 signaling pathway could have broader relevance in the landscape of pediatric sarcomas (61). But, as described above, the function of AP-1 is highly cell type and context dependent and the impact of upregulation of AP-1 expression, including the potential for enhanced oncogenic signaling, is likely to differ between cancer types (57). For example, the inactivation of c-Fos in *Trp53* mutant mice leads to the development of embryonal rhabdomyosarcoma tumors and the reexpression of c-Fos in these tumor cells causes apoptosis and cell death (62, 63). However, transgenic mice that overexpress c-Fos in bone develop osteosarcomas, whereas mice overexpressing c-Jun are normal (62). Furthermore, EWS-FLI is reported to promote cooperative binding with c-Jun and c-Fos at specific promoters, but it is unknown whether this cooperativity alters the targets of AP-1 in Ewing sarcoma cells compared with other tumor types (64, 65). Consequently, due to the pleiotropic and context-dependent functions of AP-1, we expect that the role of AP-1, and specific AP-1 subunits, will vary between cancers and will need to be carefully investigated in individual sarcoma subtypes.

We used a conditional knockout (CRISPR/Cas9) and rescue approach to target the RRM1 subunit of RNR and, thereby, avoid complications that arise from the polypharmacology of small-molecule inhibitors of RNR. However, we recognize that inhibition of a target protein is not always functionally equivalent to a reduction in the level of that protein. Indeed, targeted protein degradation technologies, including proteolysis-targeting chimeras, have highlighted critical differences between target degradation and inhibition (52). Consequently, we validated the RRM1 knockout results with gemcitabine and hydroxyurea, which are inhibitors of the RRM1 and RRM2 subunits of RNR, respectively. We also showed that HDAC inhibitors, which reduce the level of the RRM1 protein, upregulate AP-1 and downregulate c-Myc in Ewing sarcoma cells. Overall, these results support the conclusion that a reduction in RNR activity, mediated by either enzyme inhibition or reduction in RRM1/2 protein levels, results in activation of AP-1 signaling in Ewing sarcoma tumors.

The current treatment of Ewing sarcoma, which consists of cytotoxic chemotherapy in combination with surgery and/or radiation, is relatively unchanged over the past two decades and is associated with significant on- and off-treatment morbidities (66). We and others have identified that Ewing sarcoma tumors are sensitive to inhibitors of RNR and that inhibition of the ATR-CHK1 pathway, which plays a key role in orchestrating the cellular response to DNA replication stress, sensitizes Ewing sarcoma cells to RNR inhibitors (20–22, 36, 67). Notably, a clinical trial is currently underway that is testing gemcitabine in combination with a CHK1 inhibitor in patients with Ewing sarcoma tumors (NCT05275426). We expect that a more detailed understanding of the mechanism of action of RNR inhibitors will improve the clinical use and efficacy of this class of drugs, as single agents and in combination therapies, in Ewing sarcoma and other cancers. Furthermore, additional drugs that are currently used to treat Ewing sarcoma, including irinotecan and etoposide, also cause DNA replication stress, although by different mechanisms than inhibition of RNR. However, it remains to be seen whether these drugs also upregulate AP-1 signaling, or if activation of this pathway is specific to DNA replication stress caused by inhibitors of RNR (68–70).

In summary, we used a conditional knockout (CRISPR-Cas9) and rescue approach to target RRM1 and investigate the downstream signaling pathways activated by loss of this protein in Ewing sarcoma cells. Transcriptome analysis identified that loss of RRM1, or inhibition RNR function using small-molecule drugs, upregulates expression of AP-1 family members, including c-Jun and c-Fos (Fig. 6). We also identified that upregulation of these AP-1 transcription factors in Ewing sarcoma cells is dependent on SLFN11. Notably, the doxycycline-inducible expression of c-Jun and c-Fos in Ewing sarcoma cells impairs growth and downregulates the expression of c-Myc. Overall, our work provides novel mechanistic insight into the cellular response to DNA replication stress and the critical pathways that mediate the sensitivity of cancer cells to RNR inhibitors.

## Supplementary Material

Figure S1 Dependency of Ewing sarcoma cells on RRM1 and RRM2.

Figure S2 Treatment of EW8-RRM1-KO cells with prexasertib.

Figure S3 Gene set enrichment analysis.

Figure S4 Short-term gemcitabine drug treatment.

Figure S5 Gemcitabine resistant cell lines.

Figure S6 Regulation of c-Myc.

Table S1 Gene set enrichment analysis of genes up- and down-regulated in the EW8-RRM1-KO cells after removal of doxycycline.

Supplementary Excel File 1 Quantification of immunoblots.
