# Supplementary figures and images for: Activator Protein-1 (AP-1) Signaling Inhibits the Growth of Ewing Sarcoma Cells in Response to DNA Replication Stress
Source: Cancer Res Commun. 2023 Aug 17;3(8):1580–93. doi: 10.1158/2767-9764.CRC-23-0268 (PMC10434289; doi:10.1158/2767-9764.CRC-23-0268)

Supplementary Figure 1

A

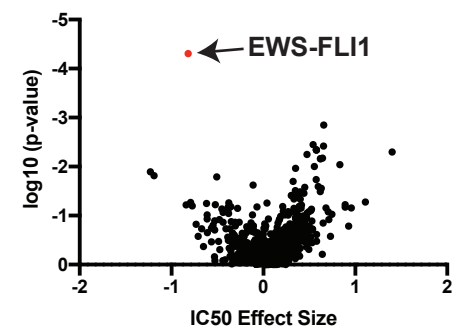

B

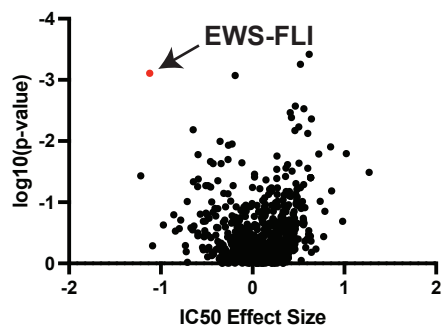

C

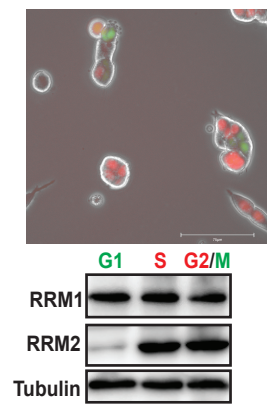

D

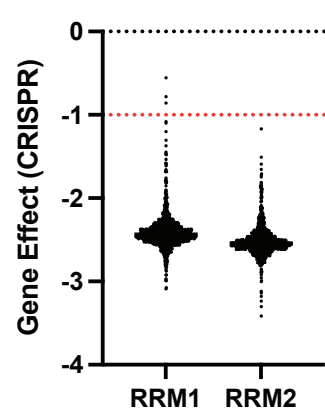

E

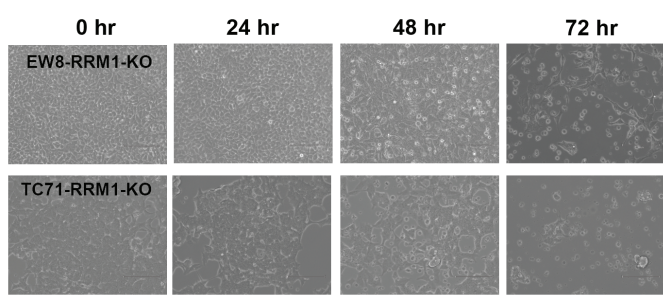

Supplement: Figure S1 — Dependency of Ewing sarcoma cells on RRM1 and RRM2. [file crc-23-0268-s01.pdf]

Supplementary Figure 2

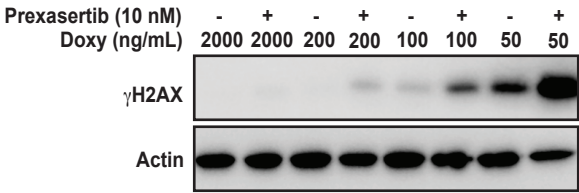

Supplement: Figure S2 — Treatment of EW8-RRM1-KO cells with prexasertib. [file crc-23-0268-s02.pdf]

Supplementary Figure 3

A

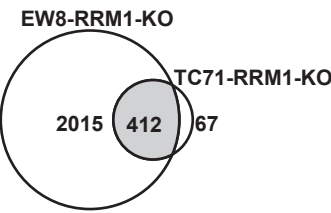

B

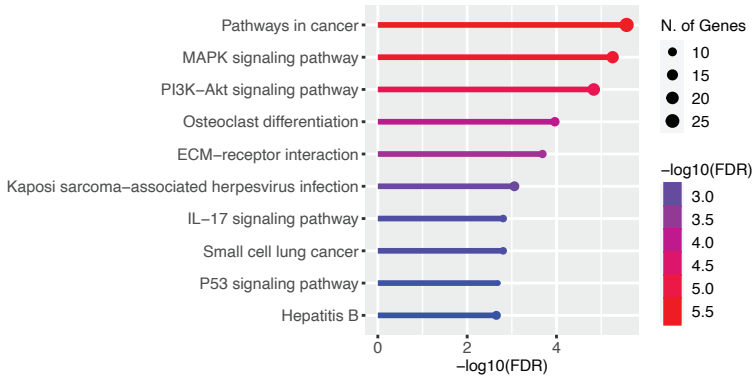

C

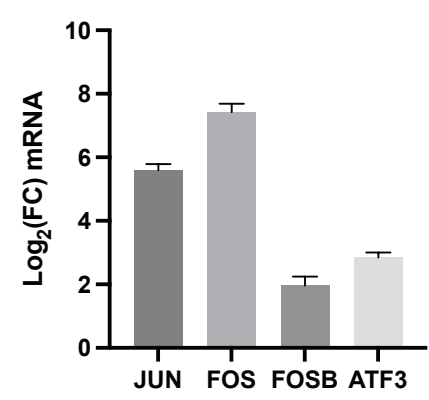

D

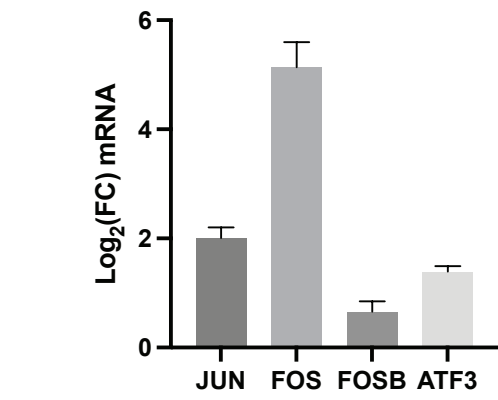

Supplement: Figure S3 — Gene set enrichment analysis. [file crc-23-0268-s03.pdf]

Supplementary Figure 4

A

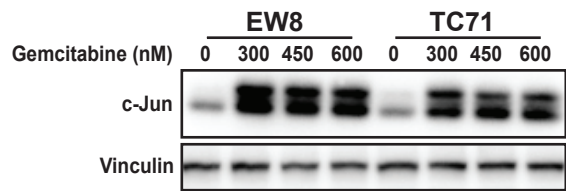

B

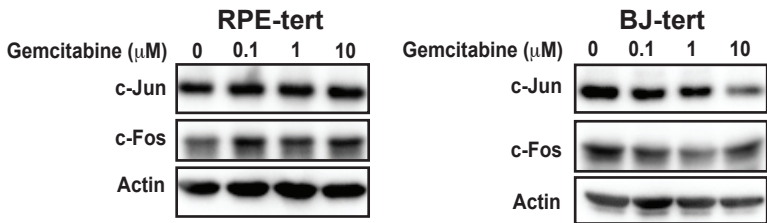

Supplement: Figure S4 — Short-term gemcitabine drug treatment. [file crc-23-0268-s04.pdf]

Supplementary Figure 5

A

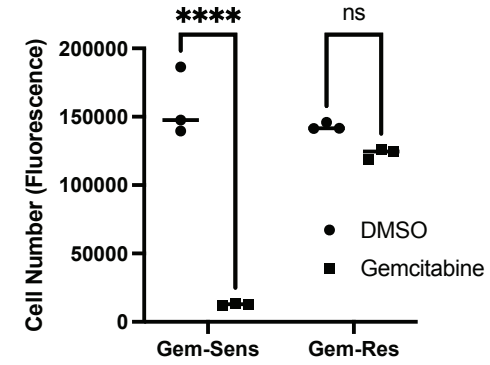

B

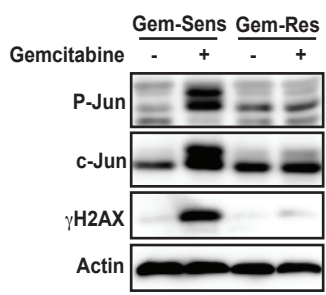

C

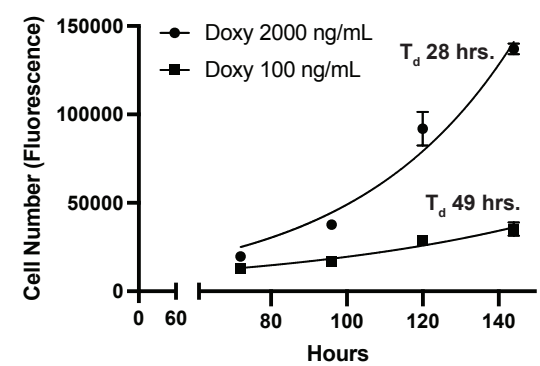

D

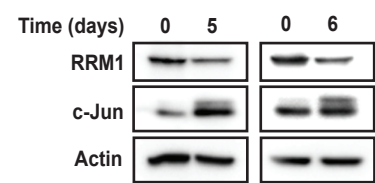

Supplement: Figure S5 — Gemcitabine resistant cell lines. [file crc-23-0268-s05.pdf]

Supplementary Figure 6

A

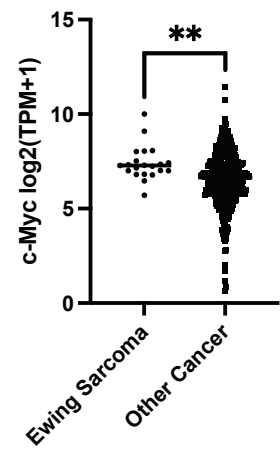

B

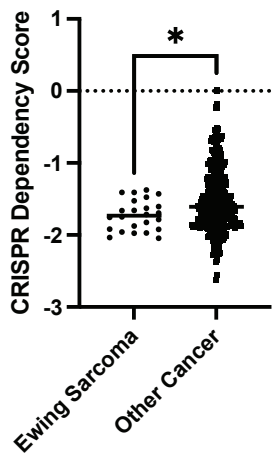

C

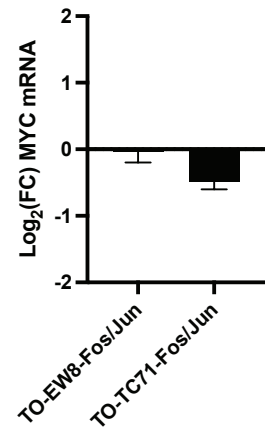

D

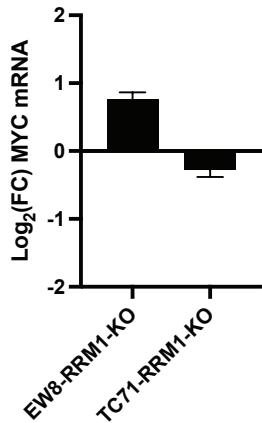

E

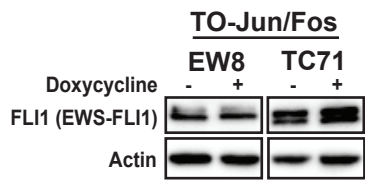

Supplement: Figure S6 — Regulation of c-Myc. [file crc-23-0268-s06.pdf]
