# Supplementary material for: Activator Protein-1 (AP-1) Signaling Inhibits the Growth of Ewing Sarcoma Cells in Response to DNA Replication Stress
Source: Cancer Res Commun. 2023 Aug 17;3(8):1580–93. doi: 10.1158/2767-9764.CRC-23-0268 (PMC10434289; doi:10.1158/2767-9764.CRC-23-0268)
Supplement: Table S1 — Gene set enrichment analysis of genes up- and down-regulated in the EW8-RRM1-KO cells after removal of doxycycline. [file crc-23-0268-s07.pdf]

Supplementary Table 1. Gene set enrichment analysis of genes up- and down-regulated in the EW8-RRM1-KO cells after removal of doxycycline and loss of RRM1 expression.

EW8-RRM1-KO			
**Direction**	**Hallmark MSigDB Pathways**	**NES**	**Adj. P-value**
Down	MYC TARGETS V1	-3.0	0.0079
Down	OXIDATIVE PHOSPHORYLATION	-2.1	0.0079
Down	MYC TARGETS V2	-1.9	0.0015
Down	E2F TARGETS	-1.7	0.0079
Down	G2M CHECKPOINT	-1.6	0.0079
Down	MTORC1 SIGNALING	-1.3	0.026
Up	TNFA SIGNALING VIA NFKB	2.2	4.70E-05
Up	INFLAMMATORY RESPONSE	2.1	4.70E-05
Up	KRAS SIGNALING UP	2.0	4.70E-05
Up	INTERFERON ALPHA RESPONSE	2.0	4.70E-05
Up	INTERFERON GAMMA RESPONSE	1.9	4.70E-05
Up	EMT	1.9	4.70E-05
Up	COAGULATION	1.9	4.70E-05
Up	IL6 JAK STAT3 SIGNALING	1.7	9.10E-05
Up	HYPOXIA	1.7	4.70E-05
Up	P53 PATHWAY	1.7	4.70E-05
Up	IL2 STAT5 SIGNALING	1.7	4.70E-05
Up	APOPTOSIS	1.6	4.70E-05
Up	COMPLEMENT	1.6	0.00035
Up	MYOGENESIS	1.6	4.00E-04
**TC71-RRM1-KO**			
Down	MYC TARGETS V1	-2.0	0.00053
Down	MYC TARGETS V2	-1.7	0.0052
Up	TNFA SIGNALING VIA NFKB	2.5	6.00E-05
Up	EMT	2.1	6.00E-05
Up	INFLAMMATORY RESPONSE	2.1	6.00E-05
Up	APOPTOSIS	2.0	6.00E-05
Up	KRAS SIGNALING UP	2.0	6.00E-05
Up	HYPOXIA	2.0	6.00E-05
Up	P53 PATHWAY	2.0	6.00E-05
Up	INTERFERON ALPHA RESPONSE	1.9	6.00E-05
Up	IL6 JAK STAT3 SIGNALING	1.9	0.00018
Up	INTERFERON GAMMA RESPONSE	1.9	6.00E-05
Up	COAGULATION	1.8	6.00E-05
Up	ALLOGRAFT REJECTION	1.8	0.00018
Up	IL2 STAT5 SIGNALING	1.8	6.00E-05
Up	COMPLEMENT	1.8	0.00015
Up	UV RESPONSE UP	1.8	0.00018
Up	XENOBIOTIC METABOLISM	1.6	0.0016
Up	KRAS SIGNALING DN	1.6	0.0072
Up	ANGIOGENESIS	1.5	0.067
